# Squamous transformation of EGFR-mutant lung adenocarcinoma after EGFR-TKI therapy: clonal continuity, tissue-based diagnosis, and therapeutic evidence gaps

**DOI:** 10.3389/fonc.2026.1910407

**Published:** 2026-08-03

**Authors:** Lijiang Zhou, Pengyan Zhang, Qiang Liu, Linlin Wang

**Affiliations:** 1 Department of Oncology, Affiliated Hospital of Liaoning University of Traditional Chinese Medicine, Shenyang, Liaoning, China; 2 Department of Thoracic Surgery, Shenyang Tenth People’s Hospital, Shenyang, Liaoning, China; 3 Oncology Department of Integrated Traditional Chinese and Western Medicine, Shenyang Tenth People’s Hospital, Shenyang, Liaoning, China

**Keywords:** EGFR-mutant lung cancer, EGFR-TKI resistance, lineage plasticity, molecular reassessment, organoids, squamous transformation, tissue rebiopsy, translational models

## Abstract

Assessment at progression after epidermal growth factor receptor tyrosine kinase inhibitor (EGFR-TKI) therapy in EGFR-mutant non-small cell lung cancer requires more than identifying a single resistance mechanism. It also requires attention to lineage plasticity, tumor heterogeneity, the pattern of progression, and tissue-based findings. In a subset of patients, rebiopsy may reveal squamous histological transformation, sometimes with retention of the founder EGFR mutation. When present, retention of the founder EGFR mutation supports a shared clonal origin but does not, by itself, determine treatment. Confirmation of squamous transformation also changes the evidentiary context, because results from histologically unselected post-EGFR-TKI populations cannot always be applied directly to patients with confirmed transformation. This review focuses on squamous transformation and related basal/squamous reprogramming detected at EGFR-TKI progression, with particular emphasis on histological and molecular reassessment. We distinguish between direct clinical evidence from histologically confirmed transformed tumors and supportive translational evidence from EGFR-mutant resistance models and related lineage-transition systems. Plasma circulating tumor DNA can identify selected resistance alterations and track molecular evolution, but it cannot define morphology or lineage identity. When transformation is suspected, repeat tissue biopsy remains central, and reassessment should integrate morphology, immunohistochemistry, and paired molecular comparison with the original tumor. Integrated pathological and molecular reassessment may also uncover coexisting actionable alterations and help clarify whether progression is driven mainly by the transformed component, residual EGFR-dependent disease, or mixed resistance. Current evidence does not support a transformation-specific treatment sequence, so management remains individualized. Future studies should combine prospective rebiopsy cohorts, paired tissue–plasma profiling, and clinically annotated models to identify predictors of transformation and develop lineage-informed treatment strategies.

## Introduction

Assessment at progression after epidermal growth factor receptor tyrosine kinase inhibitor (EGFR-TKI) therapy in EGFR-mutant non-small cell lung cancer (NSCLC) requires more than identifying a new genomic resistance alteration. Treatment decisions should consider the prior clinical course, including the duration of benefit from EGFR-TKI therapy, treatments already received, the anatomical pattern and tempo of progression, and coexisting resistance alterations. These factors remain relevant whether rebiopsy shows persistent adenocarcinoma or a transformed phenotype (1). Identifying squamous transformation does not, by itself, determine whether the same EGFR-TKI should be continued. Rather, it provides tissue-level evidence that the sampled lesion has acquired a squamous phenotype. Paired pathological and molecular assessment is then needed because morphology alone may not reliably establish whether the squamous lesion is clonally related to the original tumor (2). Confirmed transformation also limits direct extrapolation from therapeutic studies that did not report outcomes separately for histologically transformed tumors. Osimertinib and related EGFR-targeted strategies have improved outcomes across advanced disease, the adjuvant setting, and unresectable stage III EGFR-mutant NSCLC, while perioperative and combination approaches continue to expand the treatment landscape (3–8). Nevertheless, acquired resistance remains a major clinical challenge in advanced disease. Most resistance frameworks emphasize secondary EGFR alterations, MET amplification, bypass pathway activation, acquired fusions, and coexisting genomic mechanisms (9–12). Although clinically important, these mechanisms do not fully account for cases in which rebiopsy at progression also reveals a change in tumor lineage. The coexistence of lineage alteration and treatment failure does not necessarily indicate that the phenotypic change initiated resistance or was its sole cause.

Small-cell transformation is the best-characterized histological transformation pattern detected at EGFR-TKI progression in EGFR-mutant NSCLC. Representative clinical studies and recent reviews describe it as a lineage-reprogramming resistance state characterized by clonal continuity, frequent TP53/RB1 inactivation, reduced EGFR-dependent biology, and poor outcomes after transformation (13–15). Squamous transformation is less common and is supported by a much smaller body of evidence, although transformed squamous lesions may retain the original activating EGFR mutation after progression on osimertinib (16). Retention of the founder alteration supports clonal continuity with the original adenocarcinoma but should not be interpreted as proof of ongoing EGFR dependence or preserved sensitivity to EGFR inhibition. This distinction has practical implications for post-progression assessment. Imaging can define the anatomical pattern of progression, and plasma circulating tumor DNA (ctDNA) can identify selected molecular resistance alterations, but neither can establish histological transformation. When transformation is suspected, tissue-based reassessment is needed to determine the current morphology, immunophenotype, and lesion-specific molecular profile (17, 18). Recent reviews have examined lineage plasticity and histological transformation across tumor types, including different transformation patterns in lung cancer (19–21). Here, we focus specifically on histologically confirmed squamous transformation detected at progression after EGFR-TKI therapy in EGFR-mutant lung adenocarcinoma. Findings from related basal/squamous reprogramming models are considered supportive translational evidence rather than direct clinical evidence.

Squamous transformation detected at progression is temporally associated with treatment failure at the time of sampling, but this association does not establish causality. Lineage plasticity may arise through tumor-intrinsic genetic, transcriptional, or epigenetic programs and may also be shaped by external selective pressures, including therapy (22). Transformation may precede and contribute to reduced drug sensitivity, emerge under therapeutic selection, reflect the outgrowth of a pre-existing or previously unsampled squamous-prone component, or coexist with an independent genomic resistance event. These possibilities cannot currently be distinguished in individual patients. We therefore use squamous transformation primarily as a descriptive histological finding at progression and infer a causal role only when experimental manipulation of the lineage state produces a corresponding change in drug sensitivity. Throughout this review, we distinguish three categories of evidence: direct clinical evidence from histologically confirmed squamous-transformed tumors, supportive translational evidence from EGFR-mutant resistance models, and contextual evidence from broader lineage-transition systems. This distinction matters because many proposed mechanisms and therapeutic vulnerabilities have been derived from organoids, xenografts, cell-state models, or experimental lineage-transition systems and have not been repeatedly validated in clinically confirmed squamous-transformed tumors. Against this background, we synthesize the clinical and translational evidence on EGFR-TKI-associated squamous lineage plasticity, clarify why clonal continuity should not be equated with preserved EGFR dependence, and propose a reassessment-based framework for diagnostic and therapeutic interpretation.

## Clinicopathological recognition of squamous transformation at EGFR-TKI progression

Squamous transformation is a rare but clinically important tissue-defined phenotype identified in EGFR-mutant lung cancer at progression after EGFR-TKI therapy. It is usually recognized when rebiopsy of a progressing lesion reveals squamous carcinoma. In the multicenter retrospective study by Fujimoto et al., small-cell transformation was approximately four times more frequent than squamous transformation. Clinical courses and responses to subsequent treatment varied considerably, highlighting the need to interpret the rebiopsy findings in the context of the preceding treatment course (23). A recent retrospective analysis of 6,230 patients with advanced EGFR- or ALK-aberrant NSCLC identified histological transformation in 84 patients, including 11 with squamous transformation (24). These data highlight both the rarity of this phenotype and the likelihood of underestimation when tissue rebiopsy is not routinely performed. In progressing EGFR-mutant NSCLC, tissue-based reassessment should be considered when plasma genotyping is non-informative or fails to explain the observed clinical pattern of progression (25). Early studies of acquired EGFR-TKI resistance showed that resistant tumors may acquire genomic alterations, undergo histological change, or develop both (26, 27). Tissue sampling therefore remains important because plasma genotyping cannot determine morphology or lineage identity.

Pathological confirmation should integrate morphology and immunohistochemistry. Transformed lesions may show solid growth, keratinization, intercellular bridges, or nests of tumor cells with squamous differentiation; however, these features may be focal or incomplete in small biopsy specimens (28). Squamous differentiation is supported by expression of p40 and/or cytokeratin 5/6, together with reduced or absent expression of adenocarcinoma markers such as TTF-1 and Napsin A. No single marker should be interpreted in isolation; the overall morphological and immunophenotypic pattern is more informative (29). Following the identification of a squamous phenotype after EGFR-TKI treatment, the next diagnostic question is whether it represents true transformation of the original EGFR-mutant adenocarcinoma, a metachronous second primary squamous carcinoma, or an initially unsampled adenosquamous component. Paired genomic analyses have shown that squamous tumors arising after EGFR-mutant lung adenocarcinoma may retain the founder EGFR alteration and shared co-alterations, supporting clonal relatedness (30). However, clonal relatedness does not establish continued therapeutic dependence. Confirmation of clonal transformation, rather than a second primary squamous carcinoma or an unsampled adenosquamous component, therefore, relies on integrated tissue morphology, immunophenotype, and, where available, paired molecular findings.

Although squamous transformation is generally identified at EGFR-TKI progression, its association with drug resistance should be interpreted with caution. Contemporary evidence places squamous transformation within a heterogeneous spectrum of resistance-associated changes; however, clinical detection at treatment failure does not establish transformation as the direct or sole cause of resistance (31). Recent studies using patient-derived samples and preclinical models have shown that EGFR-TKI exposure can enrich drug-tolerant persister populations that survive initial therapy and provide a substrate for subsequent resistance, supporting therapy-mediated selection and adaptive cell-state change as biologically plausible processes (32). However, these findings do not establish that drug-tolerant persistence necessarily culminates in squamous transformation or resolve the causal sequence in individual patients. The tissue-based study by Schoenfeld et al. also revealed substantial genomic complexity in squamous-transformed tumors, including acquired PIK3CA alterations, chromosome 3q amplification, and FGF amplification (33). These coexisting alterations may have contributed to treatment failure, lineage change, or both. Their temporal and functional relationships with the transformed phenotype cannot be determined from the available clinical samples. The study documents squamous transformation as a clinically relevant finding at osimertinib progression but leaves its causal role unresolved.

A published case further suggests that squamous histological change can occur without prior exposure to EGFR inhibition. Le et al. described a patient with EGFR exon 19-deleted lung adenocarcinoma that recurred as squamous cell carcinoma after surgery and adjuvant chemotherapy, before any EGFR-TKI treatment. The recurrent squamous tumor retained the original EGFR exon 19 deletion, and erlotinib subsequently produced a partial response lasting six months (34). This case suggests that EGFR-TKI selection pressure may not be required for squamous histological change and that acquisition of a squamous phenotype does not invariably abolish sensitivity to EGFR inhibition. Its implications remain limited, however. A single case cannot show how often transformation precedes overt resistance or occurs while a tumor remains sensitive to a particular EGFR-TKI. Because most available clinical evidence comes from retrospective rebiopsy series obtained after recognized progression, the temporal relationship between lineage change and resistance remains unresolved (35, 36). Squamous histological change without prior EGFR-TKI exposure has therefore been reported, whereas transformation arising during continued clinical benefit from an ongoing EGFR-TKI has not been conclusively documented.

Overall, histological transformation is best viewed as a tissue-defined manifestation of an altered tumor state. Experimental models show that induced basal or squamous reprogramming can reduce EGFR-TKI sensitivity in selected settings (37, 38), but these findings cannot establish the cause of treatment failure in an individual patient. Representative clinical, pathological, and translational studies relevant to EGFR-TKI-associated squamous lineage plasticity are summarized in **Table 1**.

**Table 1:** Clinical and translational evidence relevant to EGFR-TKI-associated squamous lineage plasticity.

Study	Evidence type	Treatment/resistance context	Evidence relevant to lineage switching	Diagnostic or therapeutic implication
Roca et al., 2019 (16)	Pooled analysis with an additional case	EGFR-mutant adenocarcinoma treated with EGFR-TKIs	Summarized reported cases of adenocarcinoma-to-squamous phenotype change after EGFR-TKI therapy, with retention of baseline EGFR mutations in transformed lesions	Provides pooled case-level evidence of poor prognosis after squamous transformation identified at EGFR-TKI progression
Schoenfeld et al., 2020 (33)	Paired tumor tissue analysis at osimertinib resistance	Resistance to first-line and later-line osimertinib	Identified squamous transformation together with diverse off-target genomic alterations in tissue obtained at osimertinib progression	Documents squamous transformation as a tissue-detectable finding at osimertinib progression but does not establish whether transformation or the coexisting genomic alterations caused treatment resistance
Lin et al., 2024 (17)	Prospective study of paired tissue and liquid biopsies	Resistance after first-line EGFR-TKI treatment	Compared tissue-based and plasma-based NGS at progression	Supports complementary tissue–liquid reassessment because either approach alone may miss clinically relevant resistance alterations
Fujimoto et al., 2022 (23)	Multicenter retrospective clinical cohort	EGFR-mutant lung cancer after EGFR-TKI exposure	Evaluated histological transformations detected after EGFR-TKI treatment failure; adenocarcinoma-to-SCLC transformation was more frequent than adenocarcinoma-to-squamous transformation, with 48 and 11 cases reported, respectively	Provides cohort-level quantitative context for histological transformation, highlighting the relative rarity of squamous transformation among rebiopsy-confirmed events after EGFR-TKI resistance
Ding et al., 2026 (24)	Large single-center retrospective clinical cohort with outcome and tumor-microenvironment analyses	Advanced EGFR- or ALK-aberrant NSCLC with histological transformation after targeted therapy	Identified 84 cases of histological transformation among 6,230 patients, including 11 cases of squamous transformation	Provides recent clinical evidence for rarity, therapeutic complexity, and tissue-based characterization of transformed disease
Sequist et al., 2011 (26)	Repeat-biopsy study of genotypic and histological evolution	Acquired resistance to EGFR inhibitors	Demonstrated that EGFR-mutant lung cancers can undergo histological or phenotypic evolution, including SCLC transformation and EMT, in addition to genomic resistance changes	Provides foundational evidence supporting repeat tissue reassessment at acquired resistance
Yu et al., 2013 (27)	Large rebiopsy cohort with tissue-based resistance analysis	EGFR-mutant lung cancers undergoing biopsy at EGFR-TKI resistance	Systematically analyzed resistant tumor specimens and documented both molecular resistance mechanisms and small-cell histological transformation	Supports repeat tissue sampling for detecting histological resistance patterns that require pathological review in addition to molecular testing
Park et al., 2019 (30)	Paired genomic analysis	EGFR-mutant lung adenocarcinoma transformed to squamous carcinoma after EGFR-TKI treatment	Demonstrated retention of the initial EGFR mutation in transformed squamous lesions and identified acquired PI3K/AKT/mTOR pathway alterations	Provides key paired-genomic evidence supporting clonal continuity between the original adenocarcinoma and transformed squamous carcinoma
Chua et al., 2021 (51)	Integrative genomic, transcriptomic, and immune profiling study	T790M-negative EGFR-mutant NSCLC after first- or second-generation EGFR-TKI resistance	Identified pervasive lineage plasticity with loss of adenocarcinoma-lineage marker expression in T790M-negative resistant tumors	Associates broader lineage plasticity with T790M-negative EGFR-TKI resistance but does not establish therapeutic implications specific to overt squamous transformation
Wang et al., 2024 (37)	Integrative translational study using clinical samples and experimental models	EGFR-mutant lung cancer with EGFR-TKI resistance and adeno-to-squamous transition	Supported a functional association between adeno-to-squamous transition and reduced EGFR-TKI sensitivity and identified RAPGEF3 as a candidate vulnerability in lineage-switched cells	Provides mechanistic and preclinical evidence supporting a functional association between squamous lineage transition and reduced EGFR-TKI sensitivity
Shinozaki et al., 2025 (38)	Human tumor organoid and translational model study	EGFR-mutant lung adenocarcinoma with EGFR-TKI resistance	Identified basal-shift transformation associated with EGFR therapy resistance; NKX2–1 loss induced basal-shift transformation, while CDKN2A/B loss was associated with sensitivity to CDK4/6 inhibition	Supports basal/squamous reprogramming as a candidate resistance-associated state and suggests CDK4/6 inhibition as a potential investigational strategy in selected model contexts
Shi et al., 2025 (65)	Prospective multicenter paired tissue–plasma profiling study	First-line osimertinib failure in advanced EGFR-mutant NSCLC	Identified two adenocarcinoma-to-squamous transformations and two adenocarcinoma-to-SCLC transformations at post-progression tissue assessment	Provides prospective evidence that tissue biopsy remains necessary for detecting histological transformation after osimertinib failure

This table presents representative clinical and translational studies relevant to squamous transformation and basal/squamous lineage plasticity after EGFR-TKI therapy. Direct clinical evidence from histologically confirmed transformed tumors remains limited, while findings from resistance models and lineage-transition systems provide supportive biological context. Model-derived mechanisms and vulnerabilities should therefore not be regarded as clinically established findings in squamous-transformed EGFR-mutant NSCLC. ALK, anaplastic lymphoma kinase; CDK, cyclin-dependent kinase; EGFR, epidermal growth factor receptor; EMT, epithelial–mesenchymal transition; NGS, next-generation sequencing; NSCLC, non-small cell lung cancer; SCLC, small cell lung cancer; TKI, tyrosine kinase inhibitor.

## Clinical information added by confirmation of squamous transformation

Squamous transformation should be viewed as additional information obtained at progression rather than as a stand-alone determinant of treatment. In any patient whose disease progresses on an EGFR-TKI, treatment selection should take into account the prior therapeutic course, duration of benefit, anatomical pattern and tempo of progression, patient fitness, and coexisting resistance alterations. These factors remain relevant whether rebiopsy shows persistent adenocarcinoma or a transformed phenotype (39). After progression, continued benefit from unchanged EGFR-TKI monotherapy cannot be assumed, regardless of whether rebiopsy shows persistent adenocarcinoma or squamous transformation.

Its confirmation does, however, provide important tissue-specific information. It shows that the sampled progressing tissue contains a squamous phenotype that was not documented in the baseline adenocarcinoma and raises the need to distinguish true lineage transformation from a metachronous second primary squamous carcinoma or an initially unsampled adenosquamous component. Because the glandular and squamous components of pulmonary adenosquamous carcinoma may share a common clonal origin, retention of the founder EGFR alteration alone cannot resolve this differential diagnosis (40). Histological confirmation also limits direct extrapolation from therapeutic studies that did not separately characterize or report outcomes for transformed tumors.

**Figure 1:**
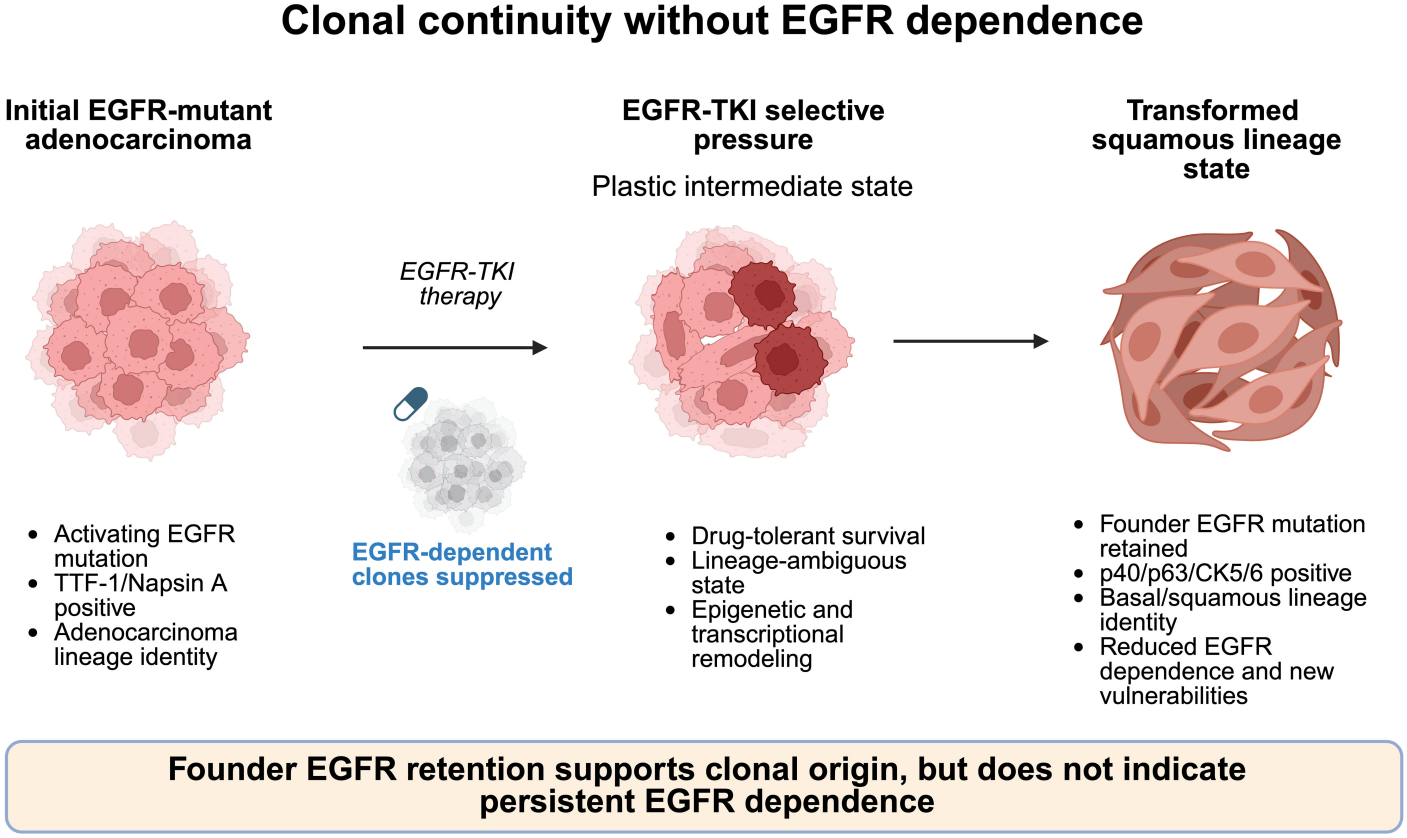
Clonal continuity and uncertain therapeutic dependence in squamous-transformed disease. A squamous-transformed lesion may retain the founder EGFR mutation and remain clonally related to the original adenocarcinoma. Mutation retention, however, does not establish continued EGFR dependence or treatment sensitivity. Its clinical significance should be interpreted in the context of the prior therapeutic course, pattern of progression, current histology, and coexisting resistance mechanisms.

Retention of the founder EGFR alteration supports clonal relatedness but does not establish current EGFR dependence. The same caution applies when rebiopsy after EGFR-TKI progression continues to show adenocarcinoma (41). Squamous transformation therefore does not automatically require discontinuation of all EGFR-directed therapy, nor does it provide sufficient grounds for continuation. The role of EGFR inhibition should instead be judged in the context of the prior treatment course, the overall pattern of progression, coexisting resistance mechanisms, and the likelihood that clinically relevant EGFR-dependent disease remains. **Figure 1** illustrates the distinction among clonal continuity, current histological identity, and therapeutic dependence after squamous transformation.

## Translational models supporting molecular and histological reassessment

Translational models are valuable in this field because clinically confirmed squamous transformation is uncommon, access to tissue samples after disease progression is often limited, and longitudinal paired samples are rare. Their evidentiary role, however, differs from that of direct clinical studies. Clinical studies show that squamous transformation can be detected after EGFR-TKI therapy and that transformed lesions may retain the founder EGFR mutation (42). Most mechanistic insights, by contrast, come from integrative profiling, resistance models, organoids, cell-state analyses, and broader lineage-transition systems (43, 44). Therefore, these findings should be interpreted as providing a biological rationale for reassessment and should be considered hypothesis-generating rather than proof of a uniform clinical mechanism.

Experimental studies suggest that EGFR-TKI therapy may suppress treatment-sensitive adenocarcinoma-like cells while allowing plastic, drug-tolerant, or lineage-ambiguous populations to persist. Such populations have been proposed as one possible substrate for basal or squamous reprogramming, whereas an alternative explanation is the selection of a pre-existing minor subclone with squamous potential. Studies on the evolution of lung cancer have identified highly plastic cell states associated with treatment resistance and divergent phenotypic potentials (45, 46). Studies on drug-tolerant persister cells further suggest that transcriptionally distinct or lineage-primed cell states may survive targeted therapy and provide a substrate for later resistance (47–50). These data support the biological plausibility of lineage plasticity; however, they do not establish whether the same sequence occurs in all patients with histologically confirmed squamous transformation.

Several studies of EGFR-mutant resistance provide relevant, although indirect, evidence. Integrative profiling of T790M-negative EGFR-mutant NSCLC demonstrated broad loss of adenocarcinoma-lineage programs in resistant tumors, suggesting that lineage transition may create therapeutic opportunities not captured by conventional genomic resistance testing (51). Comprehensive molecular characterization of clinical specimens and experimental adenocarcinoma-to-squamous transition models further implicated coordinated AKT and MYC signaling in squamous trans differentiation; however, the associated pharmacological vulnerabilities were demonstrated in preclinical systems rather than prospectively validated in patients with histologically confirmed post-EGFR-TKI squamous transformation (52). Experimental EGFR-TKI resistance models linked adeno-to-squamous transition with reduced EGFR-TKI sensitivity and identified RAPGEF3 as a candidate vulnerability in lineage-switched cells (37). In patient-derived EGFR-mutant lung adenocarcinoma organoids, a basal-shift phenotype was identified in resistant tumors without a defined genomic resistance mechanism. NKX2–1 loss promoted basal-shift transformation and reduced responsiveness to EGFR-directed therapy, whereas sensitivity to CDK4/6 inhibition was associated more specifically with CDKN2A/B loss than with basal shift alone (38). Together, these studies support reassessment of lineage identity during progression; however, their therapeutic implications require validation in clinically confirmed squamous-transformed tumors.

Squamous lineage changes may involve the loss of adenocarcinoma lineage constraints and activation of basal or squamous transcriptional programs. Reduced TTF-1/NKX2–1 and Napsin A expression may indicate erosion of adenocarcinoma identity, whereas increased expression of basal or squamous markers, including TP63/ΔNp63, SOX2, KRT5, KRT14, p40, and cytokeratin 5/6, supports the acquisition of basal or squamous features (53). NKX2–1 dosage influences enhancer accessibility, lineage dependence, and persistence during EGFR inhibition (54). Moreover, adeno-to-squamous transition is related to a ΔNp63-associated program arising through a high-plasticity intermediate state (55). Nevertheless, these markers and programs should be interpreted as part of an integrated pathological and biological assessment rather than as proof of a single mechanistic route. Epigenetic remodeling may also contribute to lineage-altered resistance states. In EGFR-mutant lung cancer models, SWI/SNF chromatin remodeling complexes promoted tyrosine kinase inhibitor resistance, whereas pharmacological interference restored osimertinib sensitivity in selected resistant models (56). Epigenetic priming is associated with resistance and oncogene amplification (57). In lineage-switching models, loss of NKX2–1 was accompanied by FoxA1/2-dependent changes in DNA methylation, enhancer activity, and chromatin organization, enabling alternative lineage programs (58). These observations demonstrate that non-genetic mechanisms may reshape tumor identity during therapy. However, the extent to which these processes initiate, maintain, or merely accompany cases of clinically confirmed squamous transformation remains unknown.

**Figure 2:**
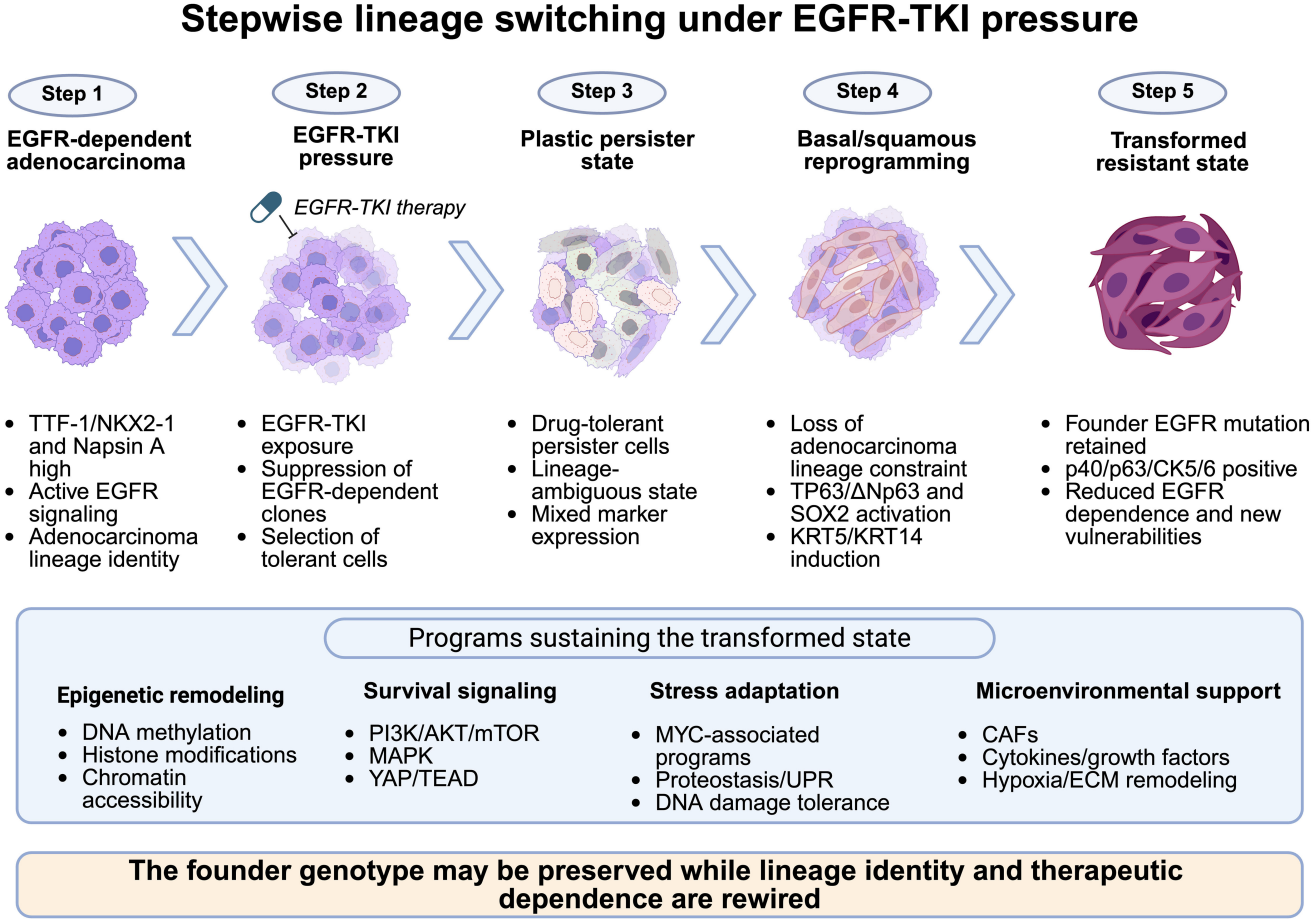
Biological rationale for molecular and histological reassessment at EGFR-TKI progression. This image summarizes model-derived processes that may occur during EGFR-TKI treatment and progression, including persistence of plastic, drug-tolerant, or lineage-ambiguous populations and subsequent basal or squamous reprogramming. Candidate programs include loss of adenocarcinoma lineage constraints, activation of basal/squamous transcriptional programs, epigenetic remodeling, adaptive survival signaling, and microenvironmental reinforcement. Their temporal order and causal relationships remain uncertain in clinically confirmed squamous-transformed tumors. The framework is therefore model-informed and hypothesis-generating and does not imply that transformation necessarily precedes or causes EGFR-TKI resistance.

Other programs may support the survival of lineage-altered populations in experimental contexts. PI3K/AKT signaling and YAP/TEAD-associated survival pathways have been implicated in osimertinib resistance and may support residual or transformed cells when EGFR signaling is no longer dominant (59–61). Lineage-specific remodeling of the tumor microenvironment has been reported in adeno-to-squamous models (62), and broader EGFR-TKI resistance studies suggest that stromal cues can influence resistant states (63). Their roles in clinically confirmed squamous transformation remain uncertain, and these mechanisms should be regarded as candidate maintenance programs rather than established clinical targets. The candidate biological programs, pathological features, and investigational implications associated with squamous lineage plasticity are summarized in **Table 2**. These findings point to several plausible, potentially overlapping explanations rather than a single clinical sequence. EGFR-TKI exposure may select pre-existing lineage-ambiguous populations, allow drug-tolerant cells to undergo further reprogramming, or coincide with genetically mediated resistance. In other cases, lineage alteration may become apparent only after resistance is established and may contribute more to maintenance than initiation of the resistant state. Current clinical evidence cannot determine which sequence applies in an individual patient. **Figure 2** therefore presents a model-informed, hypothesis-generating framework that supports integrated tissue and molecular reassessment but should not be read as a causal pathway applicable to every patient.

**Table 2:** Biological programs and investigational implications in EGFR-TKI-associated squamous lineage plasticity.

Biological program	Representative features or pathways	Relevance to squamous lineage switching	Diagnostic or pathological clues	Translational implication
Loss of adenocarcinoma lineage constraint	TTF-1/NKX2-1, Napsin A	Reduced adenocarcinoma-lineage maintenance may permit basal-shift transformation and reduced responsiveness to EGFR-directed treatment (38)	Reduced or lost TTF-1/NKX2–1 and Napsin A expression	Supports tissue-based reassessment of lineage identity at resistance
Basal/squamous transcriptional activation	TP63/ΔNp63, SOX2, p40, CK5/6, KRT5, KRT14	Activation of basal/squamous programs may support acquisition and stabilization of a squamous or basal-shift resistant state (37, 38, 55)	Increased p40, p63, CK5/6, KRT5, or KRT14 expression	Supports recognition of lineage change and investigation of lineage-associated vulnerabilities
Drug-tolerant or lineage-ambiguous intermediate states	Persister cell programs, heterogeneous cell-state adaptation	Plastic intermediate states may support survival during EGFR inhibition and provide a potential substrate for subsequent resistance-associated or lineage-altered states in experimental systems (47–50)	Resistance without an established genomic mechanism; mixed or ambiguous lineage-marker expression	Provides a rationale for studying early transitional states and plasticity-associated vulnerabilities
Epigenetic remodeling	SWI/SNF complexes, enhancer rewiring, chromatin accessibility, epigenetic priming	Chromatin-state changes may stabilize lineage-altered resistant phenotypes and enable alternative differentiation programs (54, 56–58)	Retained founder genotype with altered morphology or immunophenotype	Supports functional evaluation of chromatin-modulating strategies in lineage-switched models
Adaptive survival and microenvironmental reinforcement	PI3K/AKT signaling, YAP/TEAD programs, stromal and immune-microenvironmental cues	Adaptive survival pathways may support residual or transformed cells after EGFR-dependent populations are suppressed (59–61). Lineage-associated immune remodeling has been described in an adeno-to-squamous model (62), whereas stromal signaling has been implicated more broadly in acquired osimertinib resistance (63)	Potential coexisting pathway alterations or stromal/immune features detectable in paired tissue	Provides a rationale for biomarker-guided functional studies but does not support established pathway-directed treatment

This table summarizes candidate biological programs and investigational vulnerabilities associated with squamous lineage plasticity and related resistance states after EGFR-TKI therapy. Most supporting evidence comes from translational models, organoids, resistance systems, or broader lineage-transition studies. The listed vulnerabilities remain hypothesis-generating and have not been validated as therapeutic targets in patients with histologically confirmed squamous transformation. AKT, protein kinase B; CK5/6, cytokeratin 5/6; EGFR, epidermal growth factor receptor; KRT5, keratin 5; KRT14, keratin 14; NKX2-1, NK2 homeobox 1; PI3K, phosphoinositide 3-kinase; SOX2, SRY-box transcription factor 2; SWI/SNF, switch/sucrose non-fermentable; TEAD, TEA domain transcription factor; TKI, tyrosine kinase inhibitor; TP63, tumor protein p63; TTF-1, thyroid transcription factor 1; YAP, Yes-associated protein.

## Molecular and histological reassessment after progression on an EGFR-TKI

Assessment at progression on an EGFR-TKI should address two related questions: whether an actionable molecular resistance mechanism has emerged and whether the progressing tumor has changed histologically. Plasma ctDNA is useful for detecting selected genomic alterations and monitoring molecular evolution; however, it cannot be used to define tumor morphology, immunophenotype, or lineage identity. Therefore, tissue and liquid biopsies should be viewed as complementary rather than interchangeable when histological transformation is suspected (64). Prospective studies on osimertinib treatment failure have clarified this distinction. In a multicenter study of 149 patients with paired tissue and plasma samples collected at progression on first-line osimertinib, tissue-based analysis identified molecular resistance alterations and detected histological transformations, including adenocarcinoma-to-small-cell and adenocarcinoma-to-squamous transformations (65). Similarly, the ELIOS study prospectively compared baseline and post-progression tumor biopsies after first-line osimertinib treatment and showed that paired tissue and plasma analyses provided overlapping but non-identical information (66). These findings support the need for a reassessment strategy that combines molecular profiling and tissue-based lineage evaluation.

Representative tissue-based studies conducted after disease progression have documented histological transformation, including squamous transformation, in biopsied lesions. However, these studies were designed mainly to characterize resistance mechanisms, not to identify or prospectively validate anatomical or radiological features that predict transformation or guide biopsy-site selection (67, 68). Rapid growth, spatially discordant progression, or other atypical features should therefore not be taken as evidence that a lesion is more likely to have undergone transformation, and no lesion should be selected for biopsy on that basis alone. When tissue reassessment is performed, the findings define the lineage identity only of the sampled lesion. They should be interpreted in the context of the overall disease distribution and, where available, paired molecular findings from the original EGFR-mutant adenocarcinoma (30). Post-progression reassessment may identify actionable molecular resistance alterations while also defining the current histological state. MET amplification is a clinically relevant bypass mechanism after EGFR-TKI failure, whereas histological transformation represents a distinct tissue-defined phenotype at progression (69). Treatment decisions should therefore consider the prior therapeutic course, the overall pattern of progression, current histology, and any validated actionable alterations. Terms such as transformation-dominant, molecularly targetable, or mixed disease may help organize the clinical picture, but they should not be taken to mean that histological transformation independently drove treatment failure.

**Figure 3:**
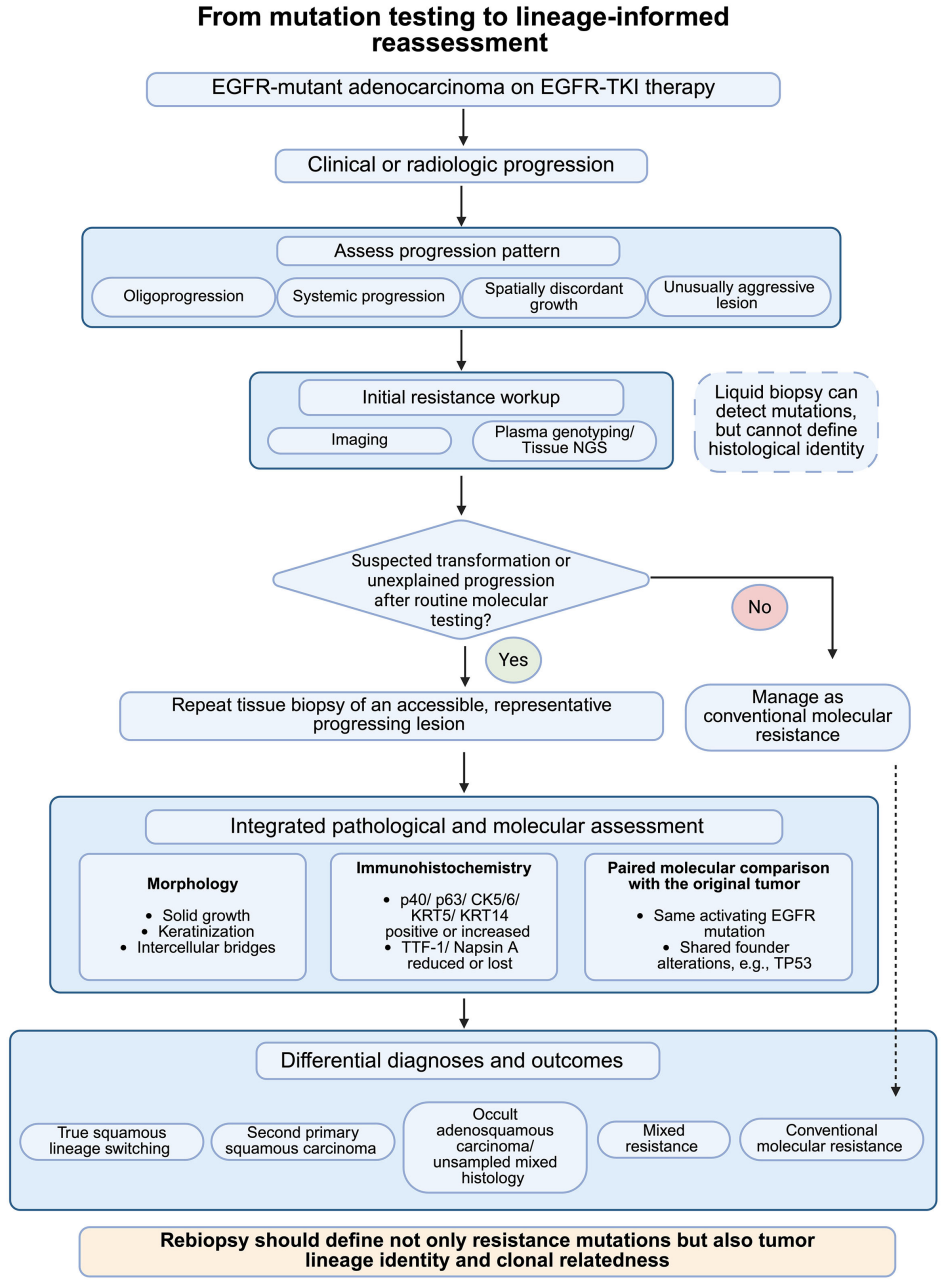
Practical diagnostic workflow for suspected squamous transformation after EGFR-TKI progression. Imaging, plasma circulating tumor DNA (ctDNA) testing, and repeat tissue biopsy provide complementary information during progression after EGFR-TKI therapy. The progression patterns shown are descriptive clinical categories and are not validated predictors of squamous transformation. Similarly, selection of an accessible and clinically representative progressing lesion reflects general biopsy practice and does not imply that any anatomical site or radiological pattern is preferentially associated with transformation. Tissue biopsy defines the morphology, immunophenotype, and lineage identity of the sampled lesion, while plasma ctDNA provides complementary genomic information. The workflow may help distinguish true squamous transformation from a second primary squamous carcinoma, an initially unsampled adenosquamous component, conventional molecular resistance, or mixed resistance.

Single-cell and spatial profiling have revealed marked intratumoral heterogeneity, diverse resistance states, and microenvironmental features associated with EGFR-TKI resistance in NSCLC (70–72). These approaches may help clarify mixed lineage states and lesion-specific biology in future studies, but they are not necessary for routine clinical diagnosis. **Figure 3** presents a practical reassessment workflow in which tissue biopsy defines lineage identity, while plasma ctDNA provides complementary genomic information when clinically appropriate.

## Limits of therapeutic evidence after histologically confirmed squamous transformation

No systemic treatment strategy has been prospectively evaluated specifically in patients with EGFR-mutant lung adenocarcinoma who develop histologically confirmed squamous transformation after EGFR-TKI therapy (73, 74). Evidence for this subgroup remains limited and comes mainly from case reports, small retrospective series, and post-progression tissue studies, with considerable variation in prior treatment, post-transformation management, and outcome reporting (75). These data are not sufficient to establish the efficacy of any particular regimen or define a validated treatment sequence after transformation. Findings from histologically unselected post-EGFR-TKI populations should therefore be viewed as contextual rather than direct evidence for this subgroup.

Management remains individualized and should take into account the prior therapeutic course, the extent and tempo of progression, the distribution of active disease, current histology, coexisting actionable alterations, and patient fitness. Continued detection of the founder EGFR alteration may support clonal continuity, but it should not determine treatment selection on its own (76). Evidence from broader EGFR-mutant or histology-defined NSCLC populations may help inform clinical decisions, although it cannot establish transformation-specific efficacy or sequencing.

**Figure 4:**
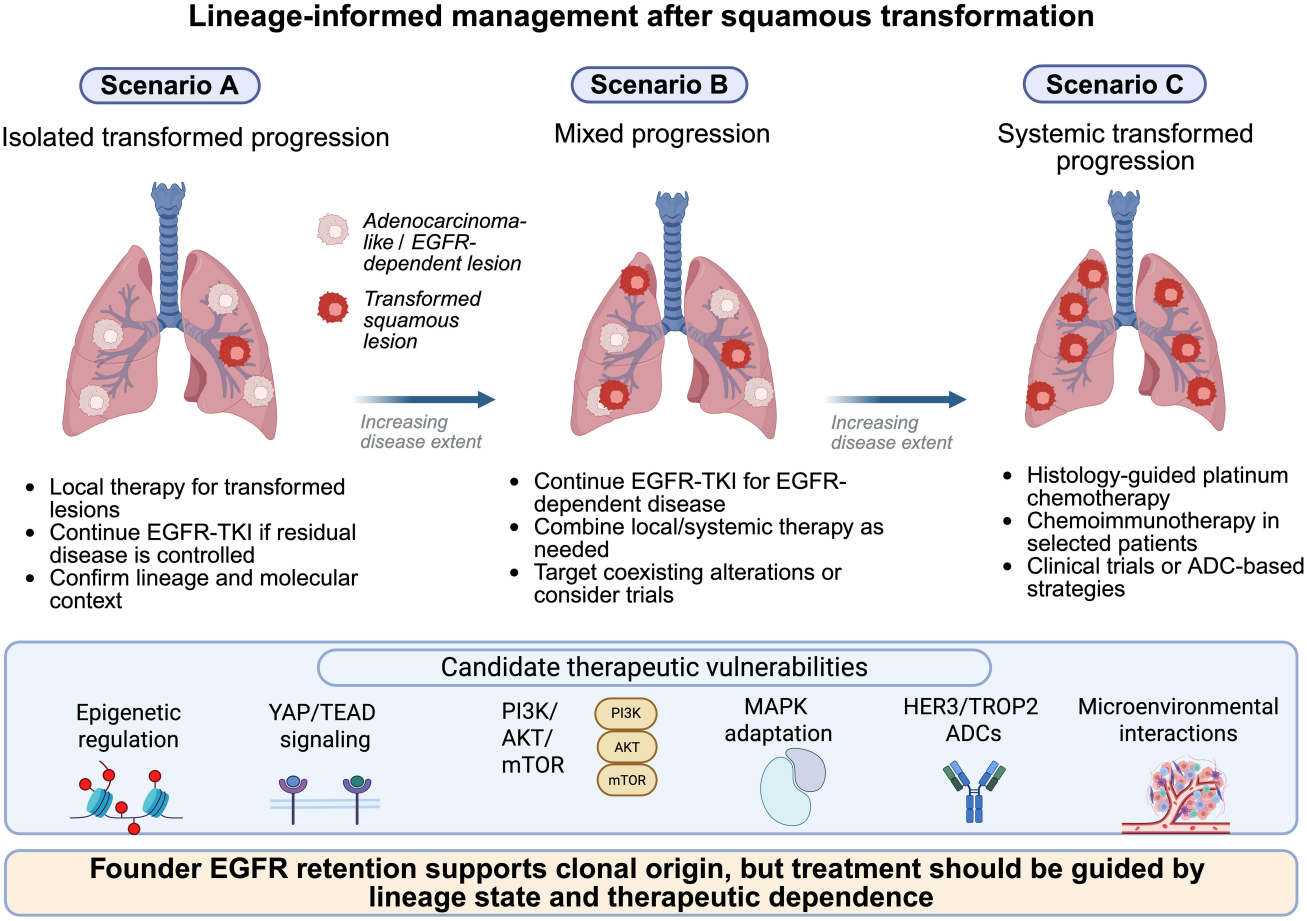
Conceptual reassessment framework after EGFR-TKI-associated squamous transformation. This image maps the clinical and molecular domains that may require reassessment after histologically confirmed squamous transformation. It is intended as a conceptual framework rather than a treatment algorithm, and inclusion of a therapeutic category does not imply transformation-specific efficacy or a recommendation for its use. Evidence from broader EGFR-mutant or histology-defined NSCLC populations may provide clinical context but cannot establish treatment efficacy or sequencing for this subgroup. Management remains individualized according to the prior therapeutic course, extent and tempo of progression, current histology, coexisting actionable alterations, and patient fitness.

Prospective registries and multicenter cohorts that report outcomes separately for histologically confirmed squamous transformation are needed before a lineage-informed treatment strategy can be defined. **Figure 4** is therefore intended only as a conceptual map of the reassessment domains and evidence gaps after transformation, not as a treatment algorithm or therapeutic recommendation.

## Conceptual synthesis: reassessing tumor identity after targeted-therapy resistance

Squamous lineage plasticity provides a useful conceptual framework for understanding a subset of lineage-altered states identified at EGFR-TKI progression; however, this framework remains incompletely validated in histologically confirmed squamous transformation. The key implication is that tumor origin and current tumor identity may diverge during therapy. Lung adenocarcinoma can undergo substantial non-genetic evolution, with transcriptional and epigenetic reprogramming contributing to morphological heterogeneity and disease progression (77). Integrated genomic and transcriptomic studies have further demonstrated that histological patterns, evolutionary history, and transcriptional states may diverge during lung cancer progression (78, 79). Translating this biological complexity into clinically actionable treatment strategies remains challenging because lineage state, molecular resistance, lesion distribution, and therapeutic vulnerability may not be concordant in individual patients.

This framework extends the interpretation of EGFR-TKI resistance beyond discrete genomic bypass mechanisms. Experimental studies in oncogene-driven lung adenocarcinoma models suggest that shifts in lineage state can alter dependence on the originally targeted pathway and contribute to resistance to targeted therapy (80, 81). Whether the same principle applies consistently to clinically confirmed squamous-transformed tumors remains unclear. Molecular continuity should therefore not be equated with preserved therapeutic dependence. Retention of the founder EGFR alteration may support a shared clonal origin, but it is not sufficient on its own to establish current treatment sensitivity (82).

Future studies should move beyond isolated post-progression snapshots and adopt longitudinal, lesion-specific approaches. TRACERx studies have shown that metastatic progression may follow different evolutionary trajectories across lesions and anatomical sites, while ctDNA-based phylogenetic analyses can help reconstruct metastatic dissemination and clonal evolution (83, 84). Although these studies did not specifically examine EGFR-TKI-associated squamous transformation, they provide a methodological basis for future work using paired pretreatment and post-transformation tissues, serial ctDNA profiling, and, where feasible, spatial technologies.

Overall, the available evidence supports viewing squamous lineage plasticity as a lineage-altered phenotype detected at EGFR-TKI progression rather than as a uniform resistance mechanism. It remains unclear whether this phenotype is induced by therapy, selected from a pre-existing population, emerges only after resistance is established, or develops alongside independent resistance mechanisms. Even when clonal continuity with the original adenocarcinoma is supported, the current histological identity and therapeutic relevance of the transformed tumor still need to be reassessed at progression.

## Challenges and future directions

Evidence on squamous lineage plasticity after EGFR-TKI therapy remains limited and methodologically heterogeneous. **Table 3** summarizes the major conclusions, the evidence supporting them, the corresponding evidence levels, and their main limitations. Recent clinical reviews and rebiopsy-based series show that most available evidence is retrospective and comes from populations selected for repeat biopsy. Histologically confirmed squamous transformation is uncommon, and the reported studies vary considerably in baseline tissue sampling, diagnostic criteria, post-transformation treatment, and outcome reporting (85, 86). These studies confirm that squamous transformation can occur, but their interpretation is limited by small sample sizes, incomplete characterization of baseline tissue, rebiopsy-related selection bias, and inconsistent clinical reporting. Mechanistic evidence comes largely from experimental resistance systems and cell-state analyses rather than from repeated validation in clinically confirmed squamous-transformed tumors (87). The true incidence, timing, causal contribution to resistance, and therapeutic implications of this phenotype therefore remain uncertain. For now, treatment considerations are best framed as individualized options guided by reassessment rather than as transformation-specific standards of care.

**Table 3:** Evidence level supporting major conclusions on EGFR-TKI-associated squamous lineage plasticity.

Major conclusion	Main supporting evidence and representative references	Evidence level	Main limitations	Interpretation
Squamous transformation can be detected at EGFR-TKI progression	Case reports, pooled analyses, retrospective cohorts, and prospective rebiopsy studies (16, 23, 24, 33, 65)	Clinical evidence, mainly retrospective with limited prospective support	Rare event; rebiopsy-selected populations; small numbers; incomplete baseline sampling; variable diagnostic criteria	Relatively established as an uncommon tissue-detectable phenotype
Squamous transformation appears less frequent than small-cell transformation	Multicenter retrospective transformation cohorts and large institutional series (23, 24)	Cohort-level clinical evidence	The reported frequency depends on rebiopsy practice, case definition, referral pattern, and biopsy-site selection	Supported, but the true incidence remains uncertain
Transformed squamous lesions may retain the founder EGFR mutation	Paired molecular analyses of original adenocarcinoma and transformed squamous lesions (16, 30, 33)	Direct paired clinical genomic evidence	Small cohorts; variable sequencing depth; limited multi-region validation	Supports clonal continuity but not preserved EGFR dependence
Plasma ctDNA cannot define histological transformation	Paired tissue–plasma profiling studies after EGFR-TKI resistance (17, 18, 65, 66)	Diagnostic evidence supported by prospective tissue–plasma studies	Tissue availability, biopsy-site bias, and spatial heterogeneity remain practical constraints	Supports tissue biopsy when transformation is suspected and clinically feasible
Basal or squamous lineage reprogramming is associated with reduced EGFR-TKI sensitivity in experimental models	Integrative profiling studies, EGFR-mutant resistance models, and organoid-based lineage-transition systems (37, 38, 51)	Translational and preclinical evidence with limited clinical validation	Limited validation in clinically confirmed squamous-transformed tumors; models may not fully recapitulate patient disease	Supports functional plausibility in experimental systems but does not establish squamous transformation as a clinical cause of resistance or determine the direction of the association
Lineage-altered resistant states may expose candidate vulnerabilities	RAPGEF3, CDK4/6, chromatin remodeling, and adaptive survival pathway studies (37, 38, 56, 59–61)	Preclinical and hypothesis-generating evidence	Candidate targets have not been prospectively validated in confirmed squamous-transformed EGFR-mutant NSCLC; some pathways are not transformation-specific	Investigational; further clinically anchored validation is needed
No standardized post-transformation treatment has been established	Reviews synthesizing published case reports and retrospective transformation cohorts (73–75)	Low-level and indirect clinical evidence	No prospective trial specific to squamous-transformed EGFR-mutant NSCLC; heterogeneous therapies, prior treatments, and outcome reporting	No established standard sequence; treatment remains individualized
Squamous histological change may occur without prior EGFR-TKI exposure	Single case report with retention of the founder EGFR mutation and subsequent response to erlotinib (34)	Isolated clinical case evidence	Single case; possible pre-existing unsampled squamous component; no serial tissue sampling or frequency estimate	Suggests that EGFR-TKI pressure is not obligatory and that squamous histology does not invariably abolish EGFR sensitivity

This table summarizes the current evidence supporting the main diagnostic, mechanistic, and therapeutic conclusions discussed in this review. Clinical evidence establishes squamous transformation as an uncommon tissue-detectable finding at EGFR-TKI progression. Most mechanistic interpretations and therapeutic implications, however, remain indirect, translational, or hypothesis-generating. ctDNA, circulating tumor DNA; EGFR, epidermal growth factor receptor; NSCLC, non-small cell lung cancer; TKI, tyrosine kinase inhibitor.

The evolutionary origin of squamous transformation is also unresolved. Plausible routes include therapy-induced lineage reprogramming, selection of a pre-existing squamous-prone subclone, expansion of an initially unsampled adenosquamous component, and progression through an adaptive or drug-tolerant intermediate state. These routes may overlap and are unlikely to be the same in every patient. Broader studies of non-genetic resistance have shown that tumor cells can pass through adaptive and drug-tolerant states during treatment, but they do not establish which of these routes, if any, leads to histologically confirmed squamous transformation (88, 89). Longitudinal collection of baseline, on-treatment, and post-progression tissues is needed to clarify when lineage divergence begins and whether it precedes radiological progression. Multiregion sampling would add further value where clinically feasible.

Future translational models should be more closely linked to clinical specimens. Patient-derived lung cancer organoid biobanks can preserve relevant histological and molecular features and have revealed lineage-state-dependent vulnerabilities, including NKX2-1-associated Wnt dependence (90). Clinically annotated lung cancer organoids have also shown potential for predicting treatment response ex vivo in patients with advanced disease (91). Neither study, however, specifically examined histologically confirmed squamous transformation after EGFR-TKI therapy, so their relevance to this subgroup remains supportive rather than direct. Whenever possible, organoids, ex vivo cultures, and xenografts should be developed from clinically annotated baseline and post-progression specimens. Computational lineage models should likewise be calibrated and validated against such data. These systems should distinguish processes that initiate or stabilize lineage change from those that sustain tumor-cell survival after transformation. Candidate vulnerabilities discussed in the preceding mechanistic sections should be functionally tested in models derived from, or benchmarked against, clinical transformation samples before they are proposed for biomarker-directed treatment.

Standardized diagnostic and clinical reporting is equally important. Future studies should document baseline histology, EGFR mutation subtype, relevant co-alterations, prior treatment sequence, the timing and anatomical site of the transformation biopsy, morphology, immunohistochemical findings, paired molecular results, subsequent treatment, and clinical outcomes. Because squamous transformation is rare, informative studies will require multicenter collaboration, harmonized data collection, and separate reporting of outcomes according to transformation phenotype. Paired tissue and plasma analyses should be incorporated when clinically feasible because they provide complementary, but not interchangeable, information (92). Single-cell and spatial approaches may help resolve cell-state heterogeneity and spatially organized resistance programs in selected research settings, but they should complement rather than replace pathological confirmation (93).

Future research should also clearly distinguish evidence from histologically confirmed squamous-transformed tumors, EGFR-mutant resistance models, and broader lineage-transition systems. Without this separation, biologically plausible mechanisms may be mistaken for clinically validated therapeutic targets. The most immediate priorities are to identify tumors at risk, determine when lineage divergence begins, develop functional models anchored to clinical samples, and test whether reassessment-guided, lineage-informed treatment strategies can improve patient outcomes.

## Conclusions

Squamous transformation after EGFR-TKI therapy is uncommon and should not be viewed as a stand-alone determinant of post-progression treatment. Prior therapy, the duration of clinical benefit, the anatomical pattern and tempo of progression, and coexisting resistance alterations remain relevant whether rebiopsy shows persistent adenocarcinoma or squamous carcinoma. Confirming squamous transformation adds several pieces of clinically relevant information: it demonstrates a squamous phenotype in the sampled progressing tissue that differs from the baseline adenocarcinoma, prompts distinction from a second primary squamous carcinoma or an initially unsampled adenosquamous component, and limits direct extrapolation from phenotype-unselected post-EGFR-TKI populations. Retention of the founder EGFR alteration supports clonal relatedness but does not prove continued EGFR dependence or, by itself, determine whether EGFR-directed therapy should remain part of subsequent treatment.

Therefore, post-progression assessment is important for distinguishing molecular history from current tumor identity. When squamous transformation is suspected, tissue-based reassessment remains central, whereas plasma ctDNA provides complementary molecular information but cannot define histological transformation. Treatment after transformation remains individualized and is best informed by the reassessed disease state rather than by EGFR mutation retention alone.

At present, most mechanistic and therapeutic implications remain translational or hypothesis-generating rather than clinically validated. Future studies should prioritize prospective rebiopsy cohorts, paired tissue–plasma analyses, standardized diagnostic reporting, and clinically annotated functional models. These efforts are needed to determine when squamous transformation begins, how it can be recognized earlier, and whether reassessment-guided, lineage-informed treatment strategies can improve outcomes.
